# Contribution of the cyclic nucleotide gated channel subunit, CNG-3, to olfactory plasticity in *Caenorhabditis elegans*

**DOI:** 10.1038/s41598-017-00126-7

**Published:** 2017-03-13

**Authors:** Damien M. O’Halloran, Svetlana Altshuler-Keylin, Xiao-Dong Zhang, Chao He, Christopher Morales-Phan, Yawei Yu, Julia A. Kaye, Chantal Brueggemann, Tsung-Yu Chen, Noelle D. L’Etoile

**Affiliations:** 1 Department of Biological Sciences, The George Washington University, Science and Engineering Hall 6000, 800 22nd St N.W., 20052 Washington DC, USA; 2 Institute for Neuroscience, The George Washington University, 636 Ross Hall, 2300 I St. N.W., 20052 Washington DC, USA; 3 UCSF Diabetes Center, Eli and Edythe Broad Center of Regeneration Medicine and Stem Cell Research, Department of Cell and Tissue Biology, University of California, 35 Medical Center Way, San Francisco, CA 94143 USA; 4 Department of Internal Medicine, Davis, 451 E Health Science Drive, Genome & Biomedical Sciences Facility, University of California, Davis, CA 95616 USA; 5 Center for Neuroscience, University of California, Davis, 1544 Newton Ct., Davis, CA 95616 USA; 6 Department of Molecular and Cell Biology, University of California, Berkeley, CA 94720 USA; 7 Gladstone Institute of Neurological Disease, University of California, San Francisco, CA 94158 USA; 8 Kavli Institute for Fundamental Neuroscience and Department of Cell and Tissue Biology, University of California, 35 Medical Center Way, San Francisco, CA 94143 USA; 9 Department of Neurology, University of California, Davis, 4860 Y Street, Sacramento, CA 95817 USA

## Abstract

In *Caenorhabditis elegans*, the AWC neurons are thought to deploy a cGMP signaling cascade in the detection of and response to AWC sensed odors. Prolonged exposure to an AWC sensed odor in the absence of food leads to reversible decreases in the animal’s attraction to that odor. This adaptation exhibits two stages referred to as short-term and long-term adaptation. Previously, the protein kinase G (PKG), EGL-4/PKG-1, was shown necessary for both stages of adaptation and phosphorylation of its target, the beta-type cyclic nucleotide gated (CNG) channel subunit, TAX-2, was implicated in the short term stage. Here we uncover a novel role for the CNG channel subunit, CNG-3, in short term adaptation. We demonstrate that CNG-3 is required in the AWC for adaptation to short (thirty minute) exposures of odor, and contains a candidate PKG phosphorylation site required to tune odor sensitivity. We also provide *in vivo* data suggesting that CNG-3 forms a complex with both TAX-2 and TAX-4 CNG channel subunits in AWC. Finally, we examine the physiology of different CNG channel subunit combinations.

## Introduction

Cyclic nucleotide gated (CNG) channels are nonselective cation channels that are gated by direct binding of cGMP or cAMP. CNG channels are composed of alpha and beta subunits, and the stoichiometry of CNG channels has been resolved for bovine photoreceptor cells, human cone photoreceptors, and rat olfactory neurons^1–4^. The presence of beta (i.e. B-type) subunits within the channel has been shown to confer enhanced Ca^2+^ permeation and modulation by Ca^2+^-calmodulin^5, 6^, as well as changes in cNMP sensitivity as compared with homomeric alpha subunit channels^7^. Furthermore, the presence of specific alpha type (i.e. A-type) subunits, such as CNGA4 in the mouse olfactory sensory neurons, can contribute more plastic capabilities to the channel^8^. Therefore, within the category of alpha-type subunits there exits both ‘primary’ alpha subunits that are required for central signal transduction, and also ‘alternate’ alpha subunits that functionally contribute more plastic capabilities. In *Caenorhabditis elegans* there is one known primary alpha CNG subunit, TAX-4 and one confirmed beta subunit, TAX-2^7, 9^. Mutant animals with genetic defects in the *tax-4* or *tax-2* genes display severe defects in olfactory responses toward odors sensed by the AWC neurons^10, 11^. There are four additional CNG channel subunits in *C. elegans*: CNG-1, CNG-2, CNG-3, and CNG-4^12–15^. Clear categorization of these subunits has been difficult using bioinformatic approaches. However, previous work has tentatively categorized CNG-1 and CNG-3 as alpha subunits^13, 16^.

Sustained exposure to AWC-sensed odors will result in decreased attraction of the animal to the exposed odor, and this change in sensitivity is termed *adaptation* ^17–19^. Adaptation requires the activity of the cGMP-dependent kinase, PKG-1/EGL-4, within the AWC sensory neurons. This kinase is involved with at least two stages of adaptation: short-term adaptation which results from exposure to an AWC sensed odor for 30 mins; and long-term adaptation which results from 60 mins of odor exposure^18–20^. Furthermore, a candidate PKG phosphorylation site within the CNG channel beta subunit, TAX-2, has been shown to be required for short-term adaptation but dispensable for long-term adaptation in AWC^19^. These data implicate the CNG channel as a determinant of short-term adaptation and suggest that EGL-4/PKG-1 may promote short-term adaptation by phosphorylating TAX-2. The finding by us and others that multiple CNG subunits, including CNG-3, are expressed in AWC^12^ ^, 44^ led us to postulate that the additional subunits may represent ‘alternate’ alpha subunits that act in a manner similar to the CNGA4 subunit in mouse olfactory neurons by contributing to adaptation responses in AWC. We show here that CNG-3 is necessary in AWC for short-term adaptation and that it is not required for long-term adaptation. We also report that short term adaptation requires CNG-3 to be expressed within the AWC and we identify a candidate PKG site within CNG-3 whose phosphorylation may decrease responsiveness to the AWC sensed odors butanone and benzaldehyde. We also report that biomolecular fluorescent complementation (BiFC) assays^21, 22^ provide *in vivo* evidence that CNG-3 can complex with TAX-2 and also TAX-4 in the CNG channel. Finally, we perform *in vitro* electrophysiology experiments to show that channel composition can dramatically alter channel gating kinetics and cGMP sensitivity. The data presented here is the first description of a gene in *C. elegans* that functions specifically in short-term (30 mins) adaptation responses, and is not required for odor detection or long-term adaptation. These results indicate that CNG-3 represents an alternate alpha subunit that may contribute to behavioral plasticity by modulating channel gating kinetics and composition.

## Results

### The alpha-type CNG channel subunit CNG-3 is necessary for short-term adaptation of the AWC sensory neuron to odor

**Figure 1:**
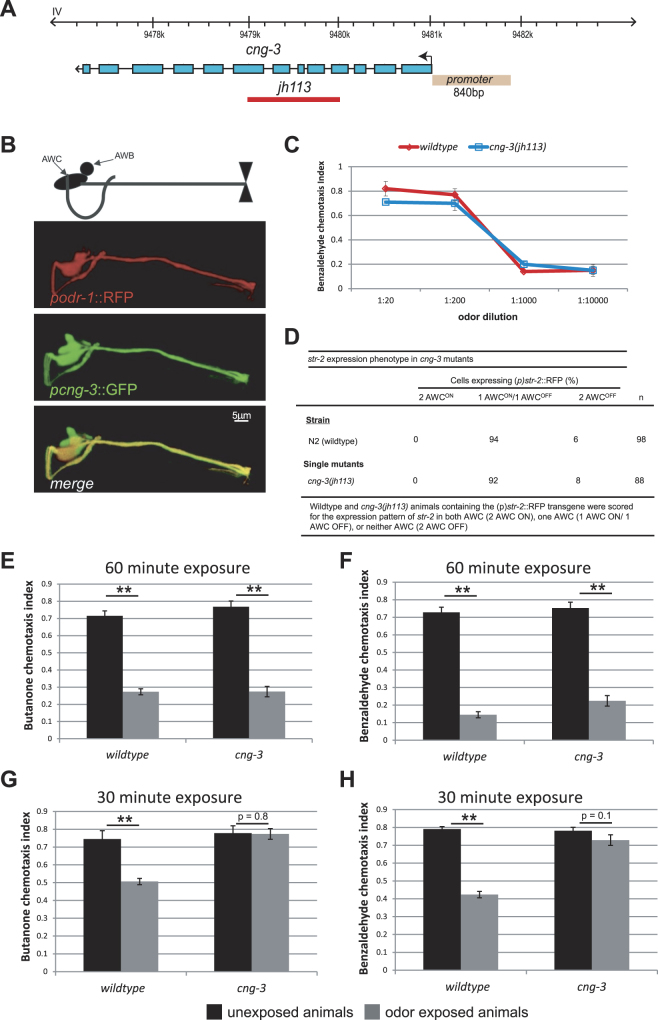
The CNG channel subunit CNG-3 is required for short-term adaptation responses in the primary sensory neuron pair AWC. (**A**) Gene structure of *cng-3* on chromosome IV indicating the position of the deletion in *jh113* ^12^. 840 bp upstream of the start site for *cng-3* was used to generate a transcriptional reporter for *cng-3*. (**B**) PCR fusion^43^ was used to fuse the promoter region of *cng-3* to GFP. In the bottom panel, an overlay of the GFP and RFP expression patterns depicts the co-expression of GFP and RFP in the AWC neuron. (**C**) Chemotaxis assays were performed for wildtype and *cng-3(jh113)* animals to various concentrations of benzaldehyde. At each concentration, *cng-3(jh113)* mutant animals performed the same as wildtype animals (paired t-test). (**D**) The G-protein coupled receptor, STR-2, is asymmetrically expressed in only one of the two AWC neurons^23, 24^. The number of wildtype animals that expressed the transcriptional reporter, *(p)str-2*::RFP in only one of the AWC neurons was compared to that of *cng-3(jh113)* mutant animals, and no significant difference was observed (*p* > 0.7 using Chi-square test or Fisher’s exact test). 94% of wildtype animals (N = 98) exhibited asymmetrical expression of the reporter and 92% of *cng-3(jh113)* mutant animals (N = 88) exhibited asymmetrical expression of the *str-2* transcriptional reporter. (**E,F**) Wildtype and *cng-3(jh113)* mutant animals were tested for an ability to adapt to the AWC sensed odor butanone (**E**) and benzaldehyde (**F**) after 60 mins odor exposure. In each case there was a significant difference in the chemotaxis index after odor exposure as compared to unexposed control animals (paired t-test, **p < 0.01). **(G–H)** Wildtype animals and *cng-3(jh113)* mutant animals were tested for an ability to adapt after 30 mins odor exposure to the AWC sensed odor butanone **(G)** and benzaldehyde **(H)**. In each case there was a significant difference in the chemotaxis index after odor exposure as compared to unexposed control cases for wildtype animals (paired t-test, **p < 0.01), however, in the case of *cng-3(jh113)* mutant animals there was no significant change in the chemotaxis index after odor exposure as compared with unexposed animals (paired t-test).

The *cng-3* mutant allele used in our study (*jh113*) has several exons of the *cng-3* gene deleted^12^ (Fig. 1A). We generated animals that should express GFP under the control of the putative *cng-3* promoter (termed *(p)cng-3)* by placing sequences 840 bp upstream of the start codon for *cng-3* in frame with the start codon of GFP (Fig. 1A). This reporter construct (*(p)cng-3*::GFP) was microinjected into wildtype animals. The resulting expression pattern of GFP driven by the *cng-3* upstream sequences was similar to that described previously^12, 13^ and indicated that *cng-3* is likely to be expressed in multiple amphid neurons including the AWC neuron pair (Fig. 1B). To investigate the consequence of the genetic deletion within the *cng-3* gene on odor taxis, we examined the ability of *cng-3(jh113)* mutant animals to migrate towards various concentrations of the AWC sensed odor benzaldehyde (Fig. 1C). We found that *cng-3(jh113)* mutant animals were as attracted to different concentrations of benzaldehyde as wildtype animals (Fig. 1C: *p* > 0.05 for each odor concentration between wildtype and *cng-3* ^−/−^ mutant animals) and also responded to butanone like wildtype animals (see Supplementary Fig. S1: *p* > 0.05 for each odor concentration between wildtype and *cng-3* ^−/−^ mutant animals).

The AWC neurons are a bilateral pair of morphologically identical head neurons that sense a variety of volatile attractive odors. However, there are molecular differences between the two AWC neurons^23, 24^, for example, the olfactory receptor, STR-2, is expressed in only one of the bilateral AWC neurons^24^ (termed the AWC ‘ON’) and butanone is sensed by this ON neuron. The expression pattern of STR-2 is used as one read-out for the AWC cellular identity. To test whether the deletion allele of *cng-3(jh113)* results in developmental defects, we examined the expression pattern of STR-2 in the *cng-3(jh113)* mutant background. Using the reporter *(p)str-2*-::RFP as a readout for STR-2 expression we observed that *(p)str-2*::RFP was only expressed in one of the AWC neurons in 94% (N = 98) of wildtype animals and 92% (N = 88) of *cng-3(jh113)* mutant animals. We failed to observe either wildtype or *cng-3(jh113)* mutant animals that expressed *(p)str-2*::RFP in both AWC neurons. Thus, we found that the expression of STR-2 was not different from that of wildtype animals, demonstrating that defects in the *cng-3* gene do not disrupt the STR-2 developmental program (Fig. 1D).

We next examined the ability of *cng-3(jh113)* mutant animals to adapt to 60 mins of odor exposure. Briefly, animals are exposed to the AWC sensed odor butanone (Fig. 1E) or benzaldehyde (Fig. 1F) for 60 mins in liquid and then challenged to migrate to a point source of the same odor. Wildtype animals will adapt to the odor after 60 minutes of exposure and so they will ignore the odor spot and while the buffer exposed (naive control) animals will continue to migrate towards the spot^19^. The *cng-3(jh113)* mutant animals performed just like wildtype animals in these assays (Fig. 1E,F: *p < *0.01 between unexposed and exposed animals). Next, we examined short-term adaptation behavior in these mutants. Short-term adaptation assays follow the same methodology used for long-term adaptation assays except that the exposure time to the AWC sensed odor is only 30 mins instead of 60 mins. We found that *cng-3(jh113)* mutant animals were defective in short-term adaptation assays to the AWC sensed odors butanone and benzaldehyde (Fig. 1G,H: *p* = 0.8 between unexposed and exposed *cng-3(jh113)* mutant animals to butanone and *p* = 0.1 between unexposed and exposed *cng-3(jh113)* mutant animals to benzaldehyde), thus revealing that CNG-3 is necessary for short-term adaptation responses in AWC but not required for long-term adaptation responses.

We also examined the ability of GFP tagged EGL-4 to translocate to the nucleus of AWC neurons after 60 mins benzaldehyde exposure in wildtype and *cng-3(jh113)* mutant animals and observed no significant difference in the number of animals presenting nuclear GFP::EGL-4 after long-term odor exposure in *cng-3(jh113)* mutants versus wildtype animals (see Supplementary Fig. S2), supporting our data that CNG-3 only contributes to the short-form of adaptation in AWC.

### CNG-3 is required in AWC to promote short-term adaptation

**Figure 2:**
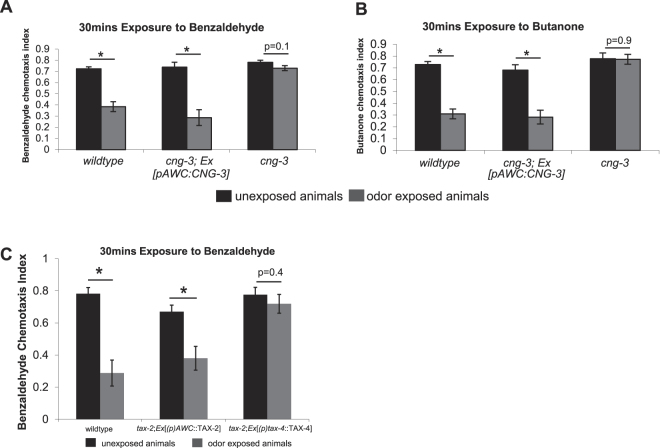
CNG-3 functions in AWC for short-term adaptation responses, and *tax-2* ^−/−^ mutants that overexpress TAX-4 are defective for short term adaptation to AWC-sensed odors. (**A**) Chemotaxis assays were performed on wildtype animals, *cng-3(jh113)* that express the cDNA encoding CNG-3 under an AWC specific promoter [*(p)AWC*::CNG-3], and also *cng-3(jh113)* mutant animals. Wildtype animals and *cng-3(jh113)* mutant animals expressing the extrachromosomal array *Ex*[*(p)AWC*::CNG-3] both exhibited significant differences in the chemotaxis index after 30 min odor exposure as compared with non-exposed animals (paired t-test, *p < 0.05). Whereas *cng-3(jh113)* mutant animals exhibited no significant change in the chemotaxis index when comparing exposed animals to that of non-exposed animals (paired t-test, *p* = 0.1). (**B**) Chemotaxis assays were performed on wildtype animals, *cng-3(jh113)*; *Ex*[*(p)AWC*::CNG-3] animals and *cng-3(jh113)* mutant animals to the AWC sensed odor butanone. Wildtype animals and *cng-3(jh113)*; *Ex*[*(p)AWC*::CNG-3] animals both exhibited significant differences in the chemotaxis index after 30 min odor exposure as compared with non-exposed animals (paired t-test, *p < 0.05), and *cng-3(jh113)* mutant animals exhibited no significant difference in chemotaxis index between exposed and non-exposed animals (paired t-test, *p* = 0.9). (**C**) Wildtype animals and also *tax-2(p691)*; *Ex*[*(p)AWC*::TAX-2 OE] transgenic animals exhibit significant differences in chemotaxis after 30 mins odor exposure as compared with unexposed controls (paired t-test, *p < 0.05). The *tax-2(p691)*; *Ex*[*(p)tax-4*::TAX-4 OE] animals can detect benzaldehyde similar to wildtype animals but fail to adapt to the odor after 30 mins of odor exposure (paired t-test, *p* = 0.4). For (**A–C**) Black bars indicate unexposed animals and gray bars indicate odor exposed animals. All assays were repeated 5 times on separate days. For all graphs, error bars represent S.E.M.

To determine if CNG-3 is required in AWC to promote adaptation to 30 minute odor exposures we expressed the cDNA encoding CNG-3 under an AWC exclusive promoter in *cng-3(jh113)* mutant animals and compared their behavior with that of wildtype and *cng-3(jh113)* mutants. We found that CNG-3 expression in AWC was sufficient to fully rescue the short-term adaptation defects of *cng-3(jh113)* mutant animals (Fig. 2A,B). Similar to wildtype animals, the transgenic animals expressing a wildtype copy of *cng-3* under an AWC promoter [*(p)AWC*::CNG-3] exhibited significant differences in their chemotaxis index when comparing benzaldehyde exposed (30 mins) to unexposed animals (Fig. 2A: *p* < 0.05–2^nd^ set of bars). This is in contrast to *cng-3(jh113)* mutant animals which failed to exhibit a significant difference in their chemotaxis index between benzaldehyde exposed (30 mins) and unexposed animals (Fig. 2A: *p* = 0.1–3^rd^ set of bars). Similar results were obtained for the AWC sensed odor butanone; we observed significant differences in the chemotaxis index of wildtype animals and transgenic animals expressing a wildtype copy of *cng-3* under an AWC promoter [*(p)AWC*::CNG-3] after butanone exposure (30 mins) as compared with unexposed animals (Fig. 2B: *p* < 0.05–1^st^ and 2^nd^ set of bars). No significant difference was observed for *cng-3(jh113)* mutant animals in their chemotaxis index between butanone exposed (30 mins) and unexposed animals (Fig. 2B: *p* = 0.9–3^rd^ set of bars). Thus, loss of the CNG-3 subunit blocks short term adaptation.

### Animals that lack the TAX-2 subunit but over express a TAX-4 subunit are defective for short term adaptation to AWC-sensed odors

Over-expressing the primary alpha subunit TAX-4 in mutants defective for the CNG channel beta subunit gene, *tax-2,* was previously shown to rescue the olfactory defects of *tax-2* ^−/−^ mutants^10^. We set out to investigate whether animals over-expressing the primary alpha subunit TAX-4 in a *tax-2(p691)* mutant background could also restore short-term adaptation responses. We overexpressed the cDNA of TAX-4 [*Ex*(*p)tax-4*::TAX-4] in a *tax-2(p691)* mutant background and examined the chemotaxis index after 30 mins exposure to benzaldehyde and compared this to unexposed animals (Fig. 2C). From these experiments we found that animals overexpressing TAX-4 in a *tax-2(p691)* mutant background did not exhibit significant differences in the chemotaxis index after 30 mins odor exposure as compared to unexposed animals (Fig. 2C, 3^rd^ set of bars: *p* = 0.4). As a control we also overexpressed the cDNA encoding the TAX-2 subunit under an AWC specific promoter, in a *tax-2* mutant background and observed significant changes in the chemotaxis index after 30 min odor exposure as compared to unexposed animals (Fig. 2C, 2^nd^ set of bars: *p* < 0.05). Thus, though over expression of TAX-4 was able to restore primary odor sensation to *tax-2* mutants, it was not able to restore short term adaptation. It is likely that the animals that over express TAX-4 have a mixture of channels comprised of TAX-4 and CNG-3 as well as some that are TAX-4 only.

### Mutation of a candidate phosphorylation site in CNG-3 interferes with short term adaptation and alters odor sensitivity

**Figure 3:**
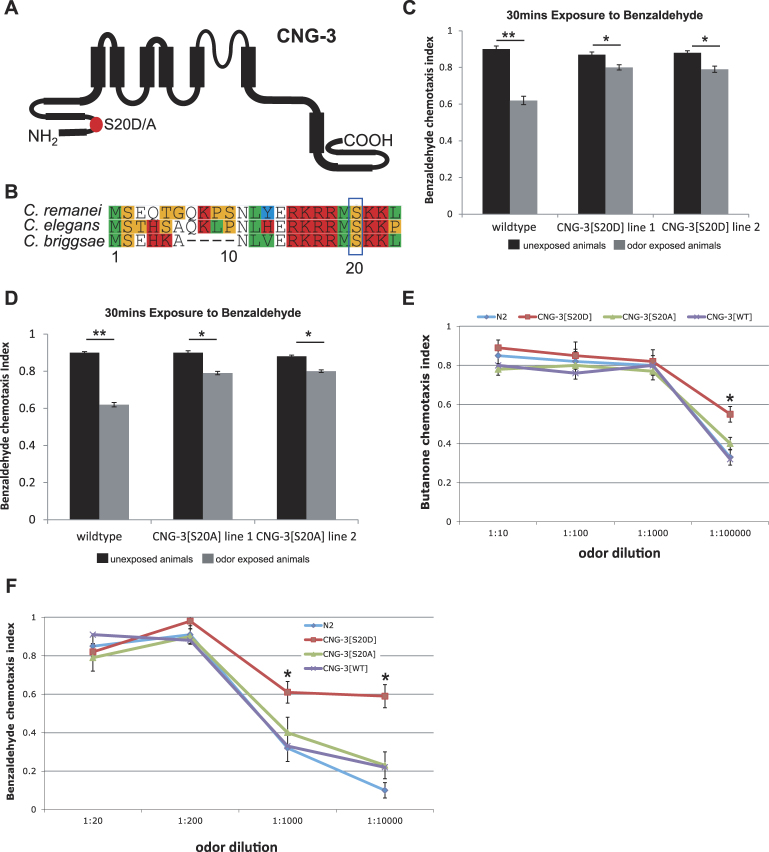
A candidate PKG site in CNG-3 is important for tuning odor sensitivity. (**A**) Cartoon structure of CNG-3 highlighting the position of the candidate PKG site at Serine 20 (S20) which was mutated to an Aspartic acid (S20D) to generate a phosphomimetic allele of CNG-3 and was mutated to an Alanine (S20A) to generate a phosphorylation defective allele of CNG-3. (**B**) Alignment of CNG-3 orthologues from *C. elegans*, *C remanei*, and *C. briggsae* reveal that this candidate PKG site is conserved. (**C**) Wildtype animals exhibited a significant change in chemotaxis index after 30 mins odor exposure as compared with unexposed animals (ANOVA with Tukey’s HSD, **p < 0.01). Transgenic animals expressing CNG-3(S20D) also exhibited a significant change in chemotaxis index after 30 mins odor exposure when compared with unexposed animals (ANOVA with Tukey’s HSD, *p < 0.05). (**D**) The chemotaxis response of two independent transgenic lines that express the phosphorylation defective version of CNG-3 (i.e. S20A) was examined and compared to wildtype responses. Wildtype animals exhibited a significant change in chemotaxis index after 30 mins odor exposure as compared with unexposed animals (ANOVA with Tukey’s HSD, *p < 0.01), and similarly the transgenic lines (line 1, 2^nd^ set of bars and line 2, 3^rd^ set of bars) expressing CNG-3(S20A) exhibited a significant change in chemotaxis index after 30 mins odor exposure when compared with unexposed animals (ANOVA with Tukey’s HSD, *p < 0.05). (**E,F**) Wildtype animals and animals expressing the normal wildtype version of CNG-3[WT], a phosphomimetic version of CNG-3(S20D) or the phosphorylation defective version of CNG-3(S20A) were tested for their ability to sense butanone (**E**) and benzaldehyde (**F**) at various concentrations. In each case the animals expressing CNG-3(S20D) exhibited a greater sensitivity to each odor at more dilute concentrations as compared with wildtype animals or animals expressing the phosphorylation defective version of CNG-3(S20A). (ANOVA with Tukey’s HSD, *p < 0.05). For all graphs, error bars represent S.E.M.

To understand the mechanism by which CNG-3 influences short-term adaptation, we searched for conserved amino acid sequences within CNG-3 that may be important for function. We identified a robust match to a candidate protein kinase G (PKG) phosphorylation site at Serine 20 (Fig. 3A). Using the NetPhos 2.0 Server^25^ the predicted score was 0.998 (maximum score is 1), and we found this candidate PKG phosphorylation site to be conserved across *C. elegans*, *C. briggsae*, and *C. remanei* (Fig. 3B). The PKG, EGL-4 has been shown to be necessary for short-term (and long-term) adaptation to AWC sensed odors^19^, and previous data implicated a candidate PKG phosphorylation site within the CNG channel subunit TAX-2 as a critical determinant of short-term adaptation^19^. To investigate the role of the candidate PKG phosphorylation site within CNG-3 in shaping short-term adaptation responses, we generated two mutant alleles: 1) the first allele mutated the Serine at position 20 to an Aspartic acid (S20D) to generate a phosphomimetic allele of CNG-3; and 2) the second allele mutated Serine 20 to an Alanine (S20A) to create a phosphorylation defective PKG site (Fig. 3A). Next we examined the ability of wildtype animals expressing the phosphomimetic version of CNG-3 (i.e. CNG-3[S20D]) to adapt to 30 mins odor exposure and compared this to wildtype animals (Fig. 3C). We generated two independent transgenic lines expressing CNG-3[S20D] and we found that each strain was able to adapt; in each case we observed significant changes in the chemotaxis index after 30 mins exposure to benzaldehyde as compared with the unexposed animals expressing CNG-3[S20D] (Fig. 3C, 2^nd^ and 3^rd^ set of bars: *p* < 0.05). Next we performed the same set of experiments but this time using transgenic animals expressing the phosphorylation defective version of CNG-3 (i.e. CNG-3[S20A]). Again, we obtained two independent transgenic lines of animals expressing CNG-3[S20A] (Fig. 3D: line 1, 2^nd^ set of bars and line 2, 3^rd^ set of bars). In each line of CNG-3[S20A] we observed a small though significant change in the chemotaxis index of animals that were exposed to benzaldehyde for 30 mins as compared with the unexposed control animals (Fig. 3D, 2^nd^ and 3^rd^ set of bars: *p* < 0.05). Interestingly, the chemotaxis response after odor exposure when compared to unexposed animals for the transgenic lines expressing CNG-3[S20D] or CNG-3[S20A] was smaller and less significant (Fig. 3C,D, 2^nd^ and 3^rd^ set of bars: *p < 0.05) when compared to the adaptation responses of wildtype animals (Fig. 3C,D, 1^st^ set of bars: **p < 0.01). Thus, in each case, the extent of adaptation is less than that of the wild type. This indicates that expressing a CNG-3 subunit that either cannot be phosphorylated or mimics phosphorylation at serine 20 creates a dominant negative channel that interferes with adaptation.

Next, we examined the chemotaxis responses of transgenic animals expressing CNG-3[S20D] or CNG-3[S20A] to various concentrations of the AWC sensed odors butanone (Fig. 3E) and benzaldehyde (Fig. 3F). In the case of animals expressing CNG-3[S20A] (line 2), we observed similar responses to that of wildtype animals and also to control animals expressing a wildtype copy of CNG-3 (CNG-3[WT]) for various concentrations of butanone (Fig. 3E: *p* > 0.05 for each dilution) and also for various concentrations of benzaldehyde (Fig. 3F: *p* > 0.05 for each dilution). Interestingly, in the case of animals expressing CNG-3[S20D] (line 1) we observed a heightened sensitivity to more dilute concentrations of odor for butanone at concentrations of 1 part per 100,000 (Fig. 3E: *p* < 0.05 for CNG-3[S20D] versus all other strains tested). Also, in the case of benzaldehyde at concentrations of 1 part per 1000 and also for 1 in 10,000 dilutions we observed significantly higher chemotaxis indices for animals expressing CNG-3[S20D] as compared with wildtype controls (Fig. 3F: *p* < 0.05 for CNG-3[S20D] expressing animals versus all other strains at 1:1000 and 1:10,000 dilutions of benzaldehyde).

### Bimolecular fluorescence complementation assays reveal an *in vivo* interaction between CNG-3 with TAX-2 and CNG-3 with TAX-4

**Figure 4:**
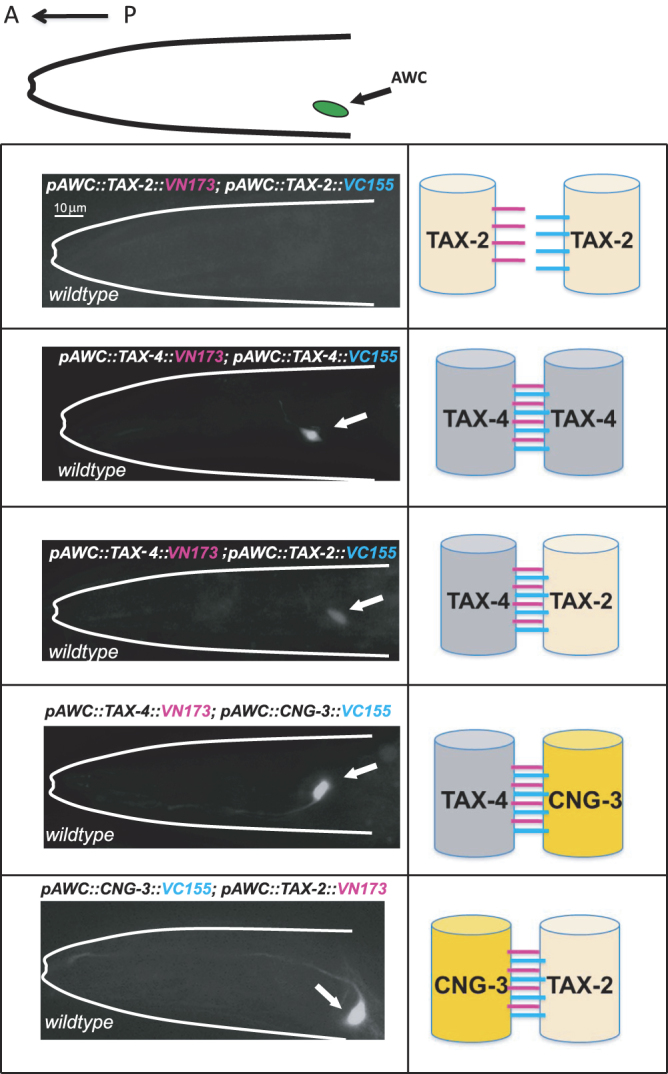
Biomolecular fluorescence complementation (BiFC) assays reveal an *in vivo* interaction between CNG-3 with TAX-2 and CNG-3 with TAX-4. The cartoon at the top illustrates the orientation of the animal for all image panels with anterior to the left and the position of the AWC neuron cell body indicated. All images were captured using a confocal microscope (Zeiss) at 63X magnification. The cartoon on the right of each image indicates the CNG channel subunit pairing that is being tested. For all BiFC experiments, the CNG channels subunit are exclusively expressed in the AWC neurons. The first microscope image tests an *in vivo* interaction between TAX-2 tagged with the N-terminal of fluorescent Venus and also TAX-2 tagged with the C-terminal of fluorescent Venus. No interaction was observed using the TAX-2::VN173 and TAX-2::VC155 pairing. Next, a similar experiment was performed using TAX-4 tagged with the N-terminal of Venus and TAX-4 tagged with the C-terminal of Venus. In this case a signal was detected in the AWC cell body indicated by a white arrow-head (39% of the transgenic animals exhibited the BiFC signal). In the next combination the pairing of TAX-4 tagged with the N-terminal of Venus and TAX-2 tagged with the C-terminal of Venus was tested, and again a signal was detected in the cell body of AWC (22% of the transgenic animals exhibited the BiFC signal). Next an *in vivo* interaction was assayed between CNG-3 tagged with the C-terminal of Venus and TAX-4 with the N-terminal of Venus, and a robust signal was observed in the cell body and dendrite of AWC (61% of the transgenic animals exhibited the BiFC signal). Finally, the pairing of CNG-3 tagged with the C terminal of Venus and TAX-2 tagged with the N-terminal of Venus was tested and again a string signal was observed in the cell body and dendrite of the AWC neuron indicated by the white arrow (43% of the transgenic animals exhibited the BiFC signal).

To probe the subunit composition of the native CNG channel in AWC, we used Biomolecular Fluorescence Complementation (BiFC) assays^21, 22, 26^ to test for an *in vivo* interaction between CNG-3 with other CNG channel subunits (Fig. 4). The basic strategy of BiFC is to split a fluorescent protein into two non-fluorescent fragments that are fused to a pair of interacting proteins. The interaction between the two proteins brings the two fragments into close proximity, allowing reconstitution of an intact fluorescent protein. The fluorescent protein being split in these experiments is Venus, and in the top panel of Fig. 4, a cartoon depicts the orientation of each of the BiFC images below as well as the position of the AWC cell body (in all images anterior is to the left). In all cases the tagged CNG channel subunit was placed under the control of a promoter that is exclusively expressed in the AWC neurons. The pair being tested is depicted in cartoon form on the right side of each BiFC microscope image. The first pair we tested was TAX-2 tagged with N-terminal of Venus and as a separate construct, TAX-2 tagged with the C terminal of Venus. These were co-expressed in the AWC neurons. In this case we failed to detect a BiFC signal. This suggests that the TAX-2 subunit is not close enough to another TAX-2 subunit to reconstitute fluorescence and thus, there may be only one TAX-2 subunit per channel.

We next expressed TAX-4 tagged with the N terminal of Venus and TAX-4 tagged with the C terminal of Venus (2^nd^ panel from top), and we observed a reconstituted fluorescent signal within the AWC cell body. This might indicate that two TAX-4 subunits are present in some AWC channels.

Next, we expressed TAX-4 tagged with the N terminal of Venus and TAX-2 tagged with the C terminal of Venus, and again we observed a BiFC signal within the cell body (3^rd^ panel from top). This indicates that TAX-2 and TAX-4 can exist in the same channel.

Next, we expressed TAX-4 tagged with the N terminal of Venus and CNG-3 tagged with the C terminal of Venus (4^th^ panel) and observed a robust BiFC signal within the cell body and also the dendrite of the AWC. Thus, TAX-4 and CNG-3 are likely to reside in the same channels.

Finally, we co-expressed CNG-3 tagged with the C terminal of Venus and TAX-2 tagged with the N terminal of Venus and observed a strong reconstituted signal in the AWC cell body and throughout the dendrite of AWC (bottom panel). This indicates that some CNG channels in AWC contain both CNG-3 and TAX-2.

We also co-expressed CNG-3 tagged with the C terminal of Venus with CNG-3 tagged with the N terminal of Venus and did not observe a BiFC signal (data not shown). This indicates that there is likely only one CNG-3 subunit per channel in AWC.

To ensure that we were closely modeling the native configuration of CNG channels within AWC, we also examined the ability of transgenic animals expressing BiFC chimeras to adapt to the AWC sensed odor benzaldehyde. In each case we found that transgenic animals expressing BiFC translational fusions were capable of detecting odor similarly to wildtype animals and adapting to the odor after sustained exposure (see Supplementary Fig. S3: *p* < 0.05 for wildtype and transgenic animals expressing BiFC chimeras before and after exposure to the AWC sensed odor benzaldehyde). In total, these experiments suggest that CNG-3 can come into complex with TAX-2 and also TAX-4 containing channels within the AWC.

### The composition of the CNG channel alters channel gating kinetics

In an attempt to understand how channel subunit composition alters odor adaptation we performed a series of *in vitro* physiology experiments. These studies were aimed at understanding how the subunit composition of the CNG channels alters channel properties. The goal was to express combinations of subunits of the *C. elegans* CNG channels in HEK cells such that they would combine to recapitulate the channels expressed in either the wildtype or short term adaptation defective *C. elegans* AWC. Thus, we hoped to compare the short term adaptation behavior in the animal with reconstituted channel physiology in cultured cells. We sought to investigate: 1) the channel properties of the wild type animal’s channel which is composed of TAX-2, TAX-4 and CNG-3; 2) the short term adaptation defective mutant strain’s TAX-4 - only channel; 3) the short term adaptation defective mutant strain’s TAX-4 and TAX-2 phosphorylation defective channel and 4) the short term adaptation defective mutant strain’s channel comprised of TAX-2 and TAX-4 only. We first examined the HEK cell response to cGMP in the absence of expressed CNG channels and found that cGMP application did not affect membrane conductance (see Supplementary Fig. S4). Thus, these cells provide a good setting for examining CNG function.

1. To explore the channel properties of the channel comprised of TAX-4, TAX-2 and CNG-3 that may represent the subunit composition of CNG channels in the AWC neurons of wild type animals, we triply transfected HEK cells with these subunits and examined the physiology of these cells (Fig. 5A). We observed a K½ of opening of the channels at 2.72 µM cGMP (Fig. 5A).

**Figure 5:**
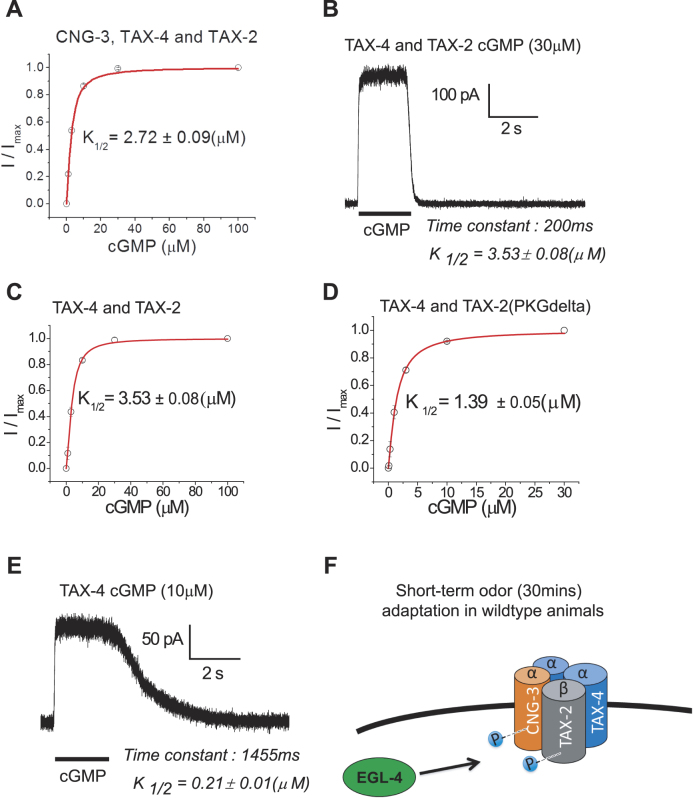
CNG channel subunit composition alters channel gating kinetics. (**A**) CNG subunits were expressed heterologously in human embryonic kidney (HEK) cells. The physiology of HEK cells triply transfected with the CNG channel subunit TAX-2, TAX-4, and CNG-3 was examined and found to exhibit a similar profile to the double transfection of TAX-4 with TAX-2. The K½ for TAX-2/TAX-4/CNG-3 transfected cells was 2.72 µM. (**B**) Next, the CNG channel subunits TAX-4 and TAX-2 were transfected into HEK cells, and cells expressing both subunits were studied using patch recordings after cGMP application. The double transfected cells exhibit fast opening and closing gating kinetics. (**C,D**) The physiology of HEK cells transfected with TAX-2 and TAX-4 (**C**) were compared with cells that were transfected with TAX-4 and a mutant allele of TAX-2 in which the candidate PKG which was previously shown to be necessary for short-term adaptation is mutated to an Alanine (**D**). The half activation concentration (K½) is reduced from 3.53 µM to 1.39 µM of cGMP. (**E**) TAX-4 exhibits a very slow closing kinetics on account of high cGMP affinity. (**F**) A model of the data is presented that suggests a native channel conformation consisting of CNG-3, TAX-2, and TAX-4. TAX-2 has previously been shown to contain a candidate PKG site important for short-term adaptation^19^ and here we reveal a candidate PKG site in CNG-3 whose phosphorylation state tunes odor sensitivity in AWC. CNG-3 and TAX-2 may represent targets of EGL-4 at possibly different time scales of the adaptation.
2. We then examined the electrophysiological properties of the channel produced by co-expression of TAX-2 and TAX-4 in HEK cells. The short term adaptation defective CNG-3 mutant is predicted to express channels with this composition and we found that the affinity for cGMP was K½ = 3.53 µM (Fig. 5B).
3. Next, we asked if the properties of the channel comprised of TAX-2 and TAX-4 would be affected if the TAX-2 subunit is unable to be phosphorylated. Previously, it was shown that mutation of a candidate PKG phosphorylation site within the CNG channel beta subunit, TAX-2, results in short-term adaptation defective animals^19^. We sought to compare the properties of channels containing wildtype TAX-2 and TAX-4 (Fig. 5B) with that of channels containing the wildtype version of TAX-4 and a version of TAX-2 in which the candidate PKG phosphorylation site has been replaced with an Alanine residue (Fig. 5D: PKG-Delta). Interestingly, here we observed an increase in cGMP affinity for channels in which the candidate PKG site was mutated. In channels containing wildtype TAX-2 and TAX-4, a K½ = 3.53 µM was observed (Fig. 5C), and in the case of channels containing the wildtype version of TAX-4 with the PKG-Delta version of TAX-2 we observed a K½ = 1.39 µM (Fig. 5D).
4. Animals that are predicted to express channels comprised of only the TAX-4 subunit are short term adaptation defective. To understand the underlying physiology of this defect we transfected HEK cells with TAX-4 cDNA under a mammalian promoter and recorded ion current from patches after cGMP stimulation (Fig. 5E). The single transfection of TAX-4 resulted in channels with an extremely high affinity for cGMP (half activation concentration K½ = 0.21 µM) that resulted in channels with rapid opening and slow closing kinetics.

Taken together these data reveal how various assemblies can alter the channel properties and suggests that in the TAX-4 only channel the gating kinetics might result in defects in short-term adaptation. Finally, we place our findings into a tentative model in Fig. 5F that illustrates a possible native channel assembly composed of TAX-4, TAX-2, and CNG-3, and depicts TAX-2 and CNG-3 as possible phosphorylation substrates of the PKG EGL-4.

Furthermore, we examined the behavior of *cng-3(jh113); tax-2(p691)* and *cng-3(jh113); tax-4(p678)* double mutants and did not observe any significant restoration of chemotaxis when compared to either single mutant (i.e. *tax-2(p691)* or *tax-4(p678)* - ANOVA with Tukey’s HSD, see Supplementary Fig. S2). It’s important to note, that the timing of the possible phosphorylation events on TAX-2 and CNG-3 by EGL-4 are likely different based on our observations that a phosphomimetic allele of CNG-3 exhibits heightened sensitivity to odor. This suggests that de-phosphorylation of CNG-3 may be an important determinant for invoking short-term adaptation. Or, that this residue is structurally important and anything you do to it to make it more negative by phosphorylation or less polar by mutation to alanine will affect the channel function.

## Discussion

Previous data has implicated the CNG channel as a key substrate for adaptation in both mammals and *C. elegans*. In mammals, rapid Ca^2+^ influx sensitizes olfactory sensory neurons (OSNs) by activating Ca^2+^ dependent Cl^−^ channels that conduct a depolarizing current. However, increased Ca^2+^ within the OSN also provides negative feedback via calmodulin to the CNG channel, resulting in adaptation^27^. In rod CNG channels, cGMP sensitivity and Ca^2+^ dependent desensitization are determined by phosphorylation on specific tyrosine residues in both the CNGA1 and CNGB1 subunits^28^. CNG channels containing only the *C. elegans* beta subunit TAX-2 and alpha subunit TAX-4 exhibit a relative ion permeability of *P*_Ca_/*P*_Na_ = 9.4^7^, which is similar to rods (*P*_Ca_/*P*_Na_ = 6.5) and much less than the cone CNG (*P*_Ca_/*P*_Na_ = 21.7). However, even though the TAX-2/TAX-4 channel is highly permeable to Ca^2+^, there is no data reporting Ca^2+^-calmodulin feedback onto CNG channels in *C. elegans*, and moreover, CNG channel subunits in *C. elegans* do not appear to contain Ca^2+^-calmodulin binding sites based on bioinformatic analysis^16, 19^. Research has shown that phosphorylation of CNG channels is however an important step for adaptation in *C. elegans*: a candidate PKG site within the beta subunit, TAX-2, has been shown to be necessary for short-term (30 mins) adaptation responses in AWC^19^, and the PKG, EGL-4, is necessary for both short-term (30 mins) and long-term (60 mins) olfactory adaptation responses within AWC^19, 20, 29, 30^. Here, we provide evidence that phosphorylation of a candidate Serine PKG site within the CNG-3 subunit (S20) sets odor sensitivity in AWC (Fig. 3). Our findings provide novel insight into the properties of neuronal plasticity in AWC, and reveal a conserved phosphorylation mechanism for neuronal desensitization from mammals to nematodes.

In vision and olfaction, the CNG channel properties vary substantially based on the subunit composition. The subunit composition and stoichiometry of the CNG channel has been shown to alter channel properties including ligand sensitivity, ion permeation, and adaptation. Furthermore, defects in CNG channel subunits can cause various diseases, especially within visual circuitry. Retinitis pigmentosa is a progressive degeneration of the retina which affects night and peripheral vision, and can be caused by mutations in the CNGA1 or CNGB1 subunits of rod photoreceptors^31, 32^. Achromatopsia, or color blindness, can be caused by defects specifically in the CNGA3 and CNGB3 subunits of cone photoreceptors^33, 34^. Within OSNs, defects within the beta subunit CNGB1 result in animals with severe olfactory defects due to defects in ciliary trafficking of the CNG channel^35, 36^. Also, the presence of the alternate alpha subunit, CNGA4, in mouse OSNs contributes a plastic capability to the animal by serving as a site for Ca^2+^-calmodulin feedback onto the CNG channel^5, 8^. Here, we extend this thesis to the nematode *C. elegans* and show that CNG channel assembly dramatically alters behavioral output by describing the first gene, *cng-3*, that contributes specifically to short-term (30 mins) adaptation, but is dispensable for primary signal transduction in AWC (Fig. 1C,E–H). We show that CNG-3 is likely an alternate alpha subunit that functions cell-autonomously within the AWC of *C. elegans* for short-term adaptation responses to the odors benzaldehyde and butanone (Fig. 2). By exploiting the dynamic properties of CNG channels, which convert chemical stimuli into electrical information, *C. elegans* can modulate odor sensitivity to prevent over-excitation and streamline foraging behavior. We also provide evidence that CNG-3 can interact with both the primary alpha subunit TAX-4 and also the beta subunit TAX-2 in *C. elegans* (Fig. 4). By examining the physiology of channels containing some or all of these subunits we observed substantial differences in the channel properties, in particular we observed a very high affinity for cGMP in homomeric TAX-4 channels, which was reduced in TAX-2/TAX-4 channels as well as TAX-2/TAX-4/CNG-3 containing channels (Fig. 5). Interestingly, in our behavioral assays we observed normal chemotaxis behavior, but short-term adaptation defects in *tax-2* ^−/−^ mutant animals wherein we overexpress the subunit TAX-4 within the AWC neurons (Fig. 2C). These data suggest that channels that can move to an open probability in response to low concentrations of cGMP do not represent a problem during primary sensory transduction in AWC, but may underlie the short-term adaptation defect during sustained odor input. In fact, integration of sustained odor input within AWC may rely on the summed ability of CNG channels to move towards a closed probability.

It is also important to note that while our data provides evidence of a possible channel assembly involving TAX-2, TAX-4, and CNG-3, previous research has revealed that distinct subpopulations of CNG channels can co-exist within individual sensory neurons in *C. elegans* ^13^. Furthermore, there are at least two other CNG channel subunits that are also expressed in the AWC neurons (CNG-2 and CNG-4)^13^. Therefore, multiple compartments that harbor distinct CNG channel assemblies likely exist within the AWC, wherein circuit output may be encoded by averaging channel activity or by summing the activity of subsets of channels that map to specific outputs. Furthermore, our *in vitro* physiology data reflects CNG channel activity in HEK cells that were cultured at 37 °C which is significantly higher that the temperature at which *C. elegans* is cultivated (typically room temperature). Addressing the question of how CNG channels localize and assemble with other subunits expressed in AWC (i.e. CNG-2 and CNG-4) will be key in understanding how the channel integrates dynamic and sustained stimulation.

## Methods

### Strains and maintenance

The following strains were used in this study: Bristol N2 wildtype, PR691 *tax-2(p691),* KJ462 *cng-3(jh113)*, KJ5561 *tax-4(p678); cng-3(jh113)*, DMH110 *tax-2(p691); cng-3(jh113)*, JZ924 N2; *Ex*[*(p)cng-3*::GFP*; (p)odr-1*::RFP*; (p)ofm-1*::GFP], JZ1004 *cng-3(jh113); Ex*[*(p)str-2:*RFP*; (p)ofm-1*::GFP], JZ1024 *cng-3(jh113); Ex*[*(p)ceh-36:CNG-3; (p)ofm-1*::GFP], JZ1274 N2; *Ex*[*(p)ceh-36*::CNG-3::VC155; *(p)ceh-36*::TAX-4::VN173*; (p)ofm-1*::GFP], JZ1320 N2; *Ex*[*(p)ceh-36*::CNG-3::VC155; *(p)ceh-36*::TAX-2::VN173; *(p)ofm-1*::GFP], JZ1266 N2; *Ex*[*(p)ceh-36*::TAX-2::VC155; *(p)ceh36*::TAX-4::VN173; *(p)ofm1*::GFP], JZ1321 N2; *Ex*[*(p)ceh36*::TAX-4::VN173; *(p)ceh36*::TAX-4::VC155; *(p)ofm-1*::GFP], JZ1122 N2; *Ex*[*(p)ceh-36*::CNG-3[S20A]; *(p)elt-2*::GFP], DMH111 *tax-2(p691)*; *Ex*[*(p)ceh-36*::TAX-2; *(p)myo-2*::RFP], JZ1125 *tax-2(p691)*; *Ex*[*(p)tax-4*::TAX-4], JZ1383 N2; *Ex*[*(p)ceh-36*::CNG-3[S20D]; *(p)elt-2*::GFP], JZ500 *pyIs500*, JZ952 *cng-3(jh113) pyIs500*. The following primer pairs were used to genotype *cng-3(jh113)* mutant animals: *cng-3 F1*: cgaagacggagttttgatcaccagctt and *cng-3 R1*: tttgtttgcaatcgctcgttgaga. Nematode Growth Media (NGM) plates were seeded with *E. coli* strain OP50 and maintained according to standard protocols^37^.

### Chemotaxis and adaptation assays

Chemotaxis and adaptation assays were performed as described previously^19, 20, 29, 30, 38–40^. Odors were diluted as follows unless otherwise stated: 1 μl benzaldehyde (Sigma) in 200 μl EtOH, 1 μl butanone (Sigma) in 1000 μl EtOH. Adaptation mixes were prepared by diluting odors as follows; 9 μl benzaldehyde into 100 ml S-Basal, 12 μl butanone into 100 ml S-Basal buffer, and populations of animals were exposed to the diluted odor for 60 minutes during long-term exposure and for 30 mins during short-term exposure. 1 µl of 1 M sodium azide was placed on top of the odor and control spots to anesthetize animals that are initially attracted to either control or odor. Animals were allowed to move for 2 hours prior to scoring. The chemotaxis index is calculated as described previously by subtracting the number of animals at the control point from the total number of animals at the odor point and dividing by the total number of animals which leave the point of origin^39^. Animals that never moved are not included in chemotaxis index calculations.

### Plasmid construction and transgenic strains

The CNG-3 rescue construct used in Fig. 2 was made by sub-cloning the CNG-3 cDNA into a plasmid containing an AWC specific promoter^41^ (*(p)ceh-36*::*GFP*, a gift from John F. Etchberger and Oliver Hobert) by using *Kpn*I and *Msc*I restriction enzymes. The resulting plasmid was injected into the germline of *cng-3(jh113)* mutant animals at 10 ng/µl alongside the co-injection marker *(p)ofm-1*::GFP at 50 ng/µl. Promoter GFP fusion^42, 43^ of *cng-3* with GFP was made using the following primers to fuse the promoter of *cng-3* with a chimera of GFP and the *unc-54* 3′UTR:

(prom)cng-3 F1:GATCTTCCTGCGAACGCCAGAAGAT

(prom)cng-3 F2:CCTGCGAACGCCAGAAGATTCCC

(prom)cng-3 F3 CTGATGTTTCTTCTACAATCTCCTCCTTATATAGCC

cng-3 fusion A: AGTCGACCTGCAGGCATGCAAGCTGAAGCCATTGGAATCCGTATATTTTC GFP F: AGCTTGCATGCCTGCAGGTCGACT

GFP R: AAGGGCCCGTACGGCCGACTAGTAGG

GFP R nest: GGAAACAGTTATGTTTGGTATATTGGG

The fusion PCR was performed as described previously^42, 43^ and injected into wildtype animals at 100 ng/µl. The GFP/*unc-54* 3′UTR fragment was PCR amplified from the plasmid pPD95.75 (AddGene). The *C. elegans* Biomolecular Fluorescent Complementation (BiFC) plasmids^26^: pCE-BiFC-VN173 and pCE-BiFC-VC155 were obtained from AddGene. The AWC specific promoter, *(p)ceh-36* was fused upstream of the cDNAs for CNG-3 and cloned into pCE-BiFC-VC155 using the restriction enzymes *Sph*I and *Kpn*I (NEB). For TAX-2, *(p)ceh-36* was fused upstream of the cDNA of TAX-2 and this fragment cloned into pCE-BiFC-VC155 using *Hind*III and *Kpn*I (NEB). For TAX-4, the same strategy was adopted as per CNG-3 above, which resulted in the *(p)ceh-36*::TAX-4 fragment cloned into each BiFC plasmid using *Sph*I and *Kpn*I (NEB). Each BiFC pairing was injected at 10 ng/µl alongside the co-injection marker *(p)ofm-1*::GFP at 50 ng/µl. For the *in vitro* studies the following plasmids were made by directionally cloning each CNG subunit cDNA into pEGFP-N1 (Clontech): *(p)cmv*::CNG-3::GFP (using *Hind*III and *BamH*I - NEB), *(p)cmv*::TAX-2::GFP (using *Kpn*I and *Sac*II - NEB) and *(p)cmv*::TAX-4::GFP (using *Kpn*I and *Xma*I - NEB). To generate the CNG-3(S20D) allele, site-directed mutagenesis (QuikChange, Stratagene) was used as per the manufacturer’s guidelines to convert the dinucleotide pair TC to GA at nucleotide positions 58 and 59 of *cng-3*. The *(p)ceh-36*::CNG-3(S20D) plasmid was injected into wildtype (N2) animals at 10 ng/µl. To generate the CNG-3(S20A) allele, site-directed mutagenesis (QuikChange, Stratagene) was also used as per the manufacturer’s guidelines to convert the T nucleotide at position 58 to a G, and the mutated plasmid injected into wildtype (N2) animals at 10 ng/µl.

### Cell culture and electrophysiology

Human embryonic kidney (HEK) cells were maintained in Dulbecco’s modified eagle medium (DMEM) supplemented with 10% heat inactivated fetal bovine serum, and 100 μg/ml penicillin/streptomycin mixture at 37 °C in an atmosphere of 5% CO_2_. Cells were transiently transfected using Lipofectamine 2000 (Invitrogen) following the manufacturers guidelines in 12-well plates. Inside-out patch recordings were performed at room temperature using Axopatch 200B, Digidata 1320 and pClamp 8.0. Pipette and bath solution used was: 130 mM NaCl, 5 mM KCl, 5 mM Hepes, 1 mM EDTA. pH = 7.4 by NaOH. Fast solution exchange was achieved by using SF-77 solution exchanger (Warner Instruments). Activation curve and half-activation concentration (K_1/2_) were obtained by fitting the data using Hill equation.

### Statistics

For all behavioral assays, between 50 and 200 animals were included, and each assay was repeated on separate days between three and five times. Statistical significance was determined using two-tailed t-tests or one-way ANOVA with post-hoc Tukey Honestly Significant Difference (HSD) tests. In the case of Fig. 1D, Chi-square and Fisher’s exact tests were performed. For each Figure the test is described in the legend. *p* < 0.05 was considered significant and denoted by a single asterisk. *p* < 0.01 was denoted by a double asterisk.

## Electronic supplementary material

Supplementary Figures
