# Supplementary material for: Contribution of the cyclic nucleotide gated channel subunit, CNG-3, to olfactory plasticity in Caenorhabditis elegans
Source: Sci Rep. 2017 Mar 13;7:169. doi: 10.1038/s41598-017-00126-7 (PMC5427828; doi:10.1038/s41598-017-00126-7)

SUPPLEMENTARY DATA FOR:

Contribution of the cyclic nucleotide gated channel subunit, CNG-3, to olfactory plasticity in *Caenorhabditis elegans*

Damien M. O'Halloran*[1,2], Svetlana Altshuler-Keylin[3], Xiao-Dong Zhang[4], Chao He[1,2], Christopher Morales-Phan[5], Yawei Yu[6], Julia A. Kaye[7], Chantal Brueggemann[8], Tsung-Yu Chen[5,9] and Noelle D. L'Etoile*[8]

1. Department of Biological Sciences, The George Washington University, Science and Engineering Hall 600, 800 22nd St N.W., Washington DC 20052
2. Institute for Neuroscience, The George Washington University, 636 Ross Hall, 2300 I St. N.W., Washington DC 20052
3. UCSF Diabetes Center, Eli and Edythe Broad Center of Regeneration Medicine and Stem Cell Research, Department of Cell and Tissue Biology, University of California, San Francisco, 35 Medical Center Way, CA 94143
4. Department of Internal Medicine, University of California, Davis, 451 E Health Science Drive, Genome & Biomedical Sciences Facility, Davis, CA 95616
5. Center for Neuroscience, University of California, Davis, 1544 Newton Ct., Davis, CA 95616
6. Department of Molecular and Cell Biology, University of California, Berkeley, CA 94720
7. Gladstone Institute of Neurological Disease, University of California, San Francisco, San Francisco, CA 94158

8. Kavli Institute for Fundamental Neuroscience and Department of Cell and Tissue Biology, University of California, San Francisco, 35 Medical Center Way, CA 94143

9. Department of Neurology, University of California, Davis, 4860 Y Street, Sacramento, CA 95817

*N.D.L. and D.O'H are co-corresponding authors, therefore, correspondence should be addressed to:

Noelle D. L'Etoile, Kavli Institute for Fundamental Neuroscience, Department of Cell and Tissue Biology, UCSF, 513 Parnassus Avenue HSW 717 UCSF San Francisco, CA 94143-0512, USA. Tel: 415-502-0311; email: noelle.letoile@ucsf.edu

Damien O'Halloran, Department of Biological Sciences and Institute for Neuroscience, The George Washington University, 636 Ross Hall, 2300 I Street N.W., Washington D.C., 20052, USA. Tel: 202-994-8955; Fax: 202-994-6100; email: damienoh@gwu.edu

Figure S1. Wildtype and mutant *cng-3(jh113)* animals exhibit normal chemotaxis responses to the AWC sensed odor butanone at various concentrations. At each concentration, *cng-3(jh113)* mutant animals performed the same as wildtype animals. For all behavioral experiments between 3 and 5 independent replicates were performed on separate days, with between 50 and 200 animals used per individual assay. Significance was compared between wildtype and mutant animals for each odor concentration (paired t-test). Error bars represent S.E.M.

Figure S2. GFP::EGL-4 nuclear translocation assays in *cng-3(jh113)* mutant animals, and behavioral analysis of *cng-3(jh113);tax-2(p691)* and *cng-3(jh113);tax-4(p678)* double mutants (A) *cng-3(jh113)* mutant animals exhibit normal nuclear translocation of GFP tagged EGL-4 after 60mins exposure to the AWC sensed odor benzaldehyde as compared with wildtype animals (ANOVA with Tukey's HSD, $^{**}p < 0.01$). Error bars represent S.E.M. **(B)** *cng-3(jh113);tax-2(p691)* and *cng-3(jh113);tax-4(p678)* double mutant animals exhibit similar chemotaxis defects to *tax-2(691)* and *tax-4(p678)* single mutants respectively (ANOVA with Tukey's HSD, $^{**}p < 0.01$).

Figure S3. Transgenic animals used in BiFC experiments exhibit normal adaptation responses. (A) Chemotaxis assays were performed on wildtype animals and animals expressing TAX-2 tagged with the N-terminal of fluorescent Venus and CNG-3 tagged with the C-terminal of fluorescent Venus. In each case, animals exhibited significant changes in their chemotaxis index after 30mins odor exposure as compared with unexposed animals (paired t-test, $*{:}p < 0.05$). **(B)** Chemotaxis was examined for wildtype animals and animals expressing TAX-4 tagged with the N-terminal of Venus and TAX-2 tagged with the C-terminal of Venus. Each

group exhibited significant changes in their chemotaxis index after 30mins odor exposure as compared with unexposed animals (paired t-test, *:$p < 0.05$). **(C)** Chemotaxis assays were performed on wildtype animals and animals expressing TAX-4 tagged with the N-terminal Venus and TAX-4 tagged with the C-terminal Venus. In each case animals' exhibited significant changes in their chemotaxis index after 30mins odor exposure as compared with unexposed animals (paired t-test, *:$p < 0.05$). **(D)** Chemotaxis assays were performed on wildtype animals and animals expressing TAX-4 tagged with the N-terminal Venus and CNG-3 tagged with the C-terminal Venus. In each case animals' exhibited significant changes in their chemotaxis index after 30mins odor exposure as compared with unexposed animals (paired t-test, *:$p < 0.05$). Error bars represent S.E.M for all graphs.

Figure S4. Non-transfected HEK cells do not exhibit activity after cGMP stimulation. When cGMP is applied to a patch from non-transfected HEK cells there is no activity response detected.

Figure S1

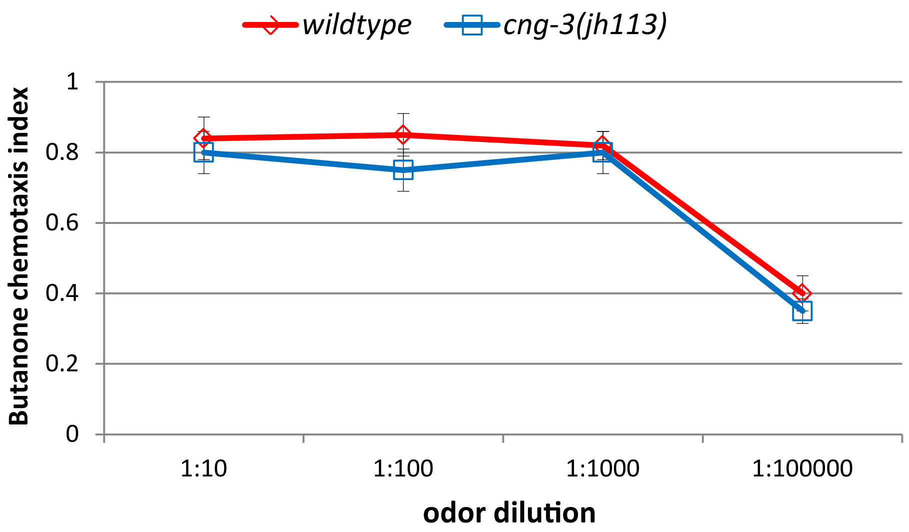

wildtype
cng-3(jh113)
Butanone chemotaxis index
1
0.8
0.6
0.4
0.2
0
1:10
1:100
1:1000
1:100000
odor dilution

Figure S2

A

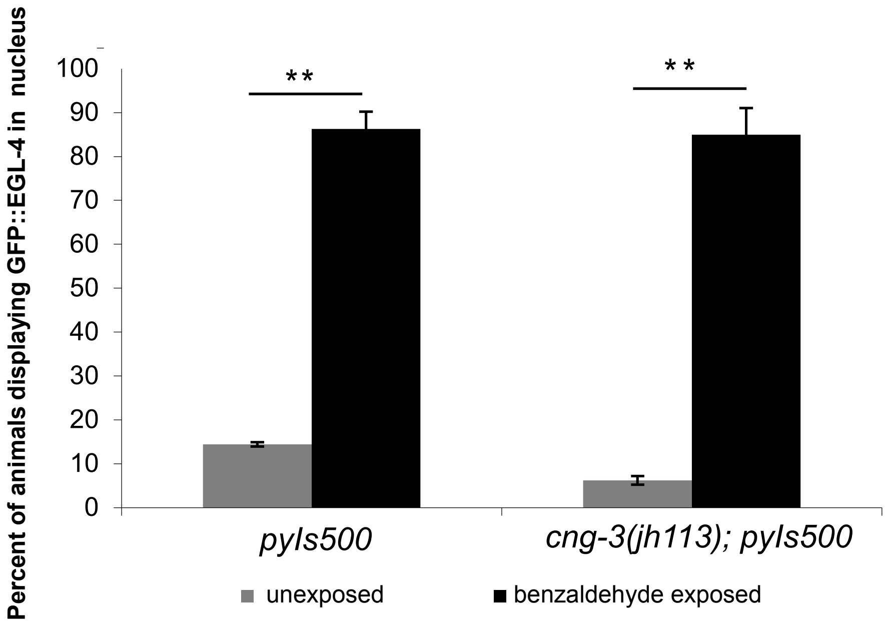

Percent of animals displaying GFP::EGL-4 in nucleus
100
90
80
70
60
50
40
30
20
10
0
**
**
pyIs500
cng-3(jh113); pyIs500
unexposed
benzaldehyde exposed

B

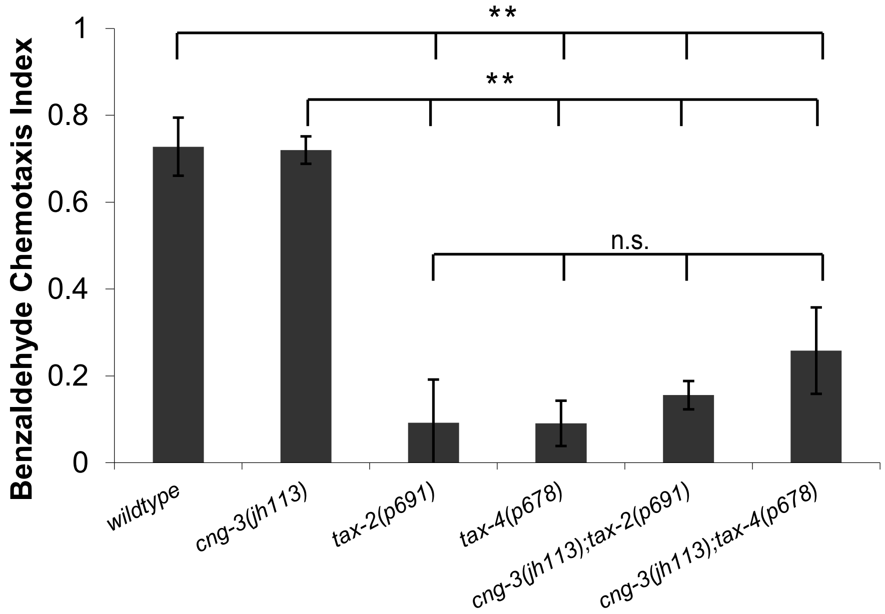

Benzaldehyde Chemotaxis Index
1
0.8
0.6
0.4
0.2
0
**
**
n.s.
wildtype
cng-3(jh113)
tax-2(p691)
tax-4(p678)
cng-3(jh113);tax-2(p691)
cng-3(jh113);tax-4(p678)

Figure S3

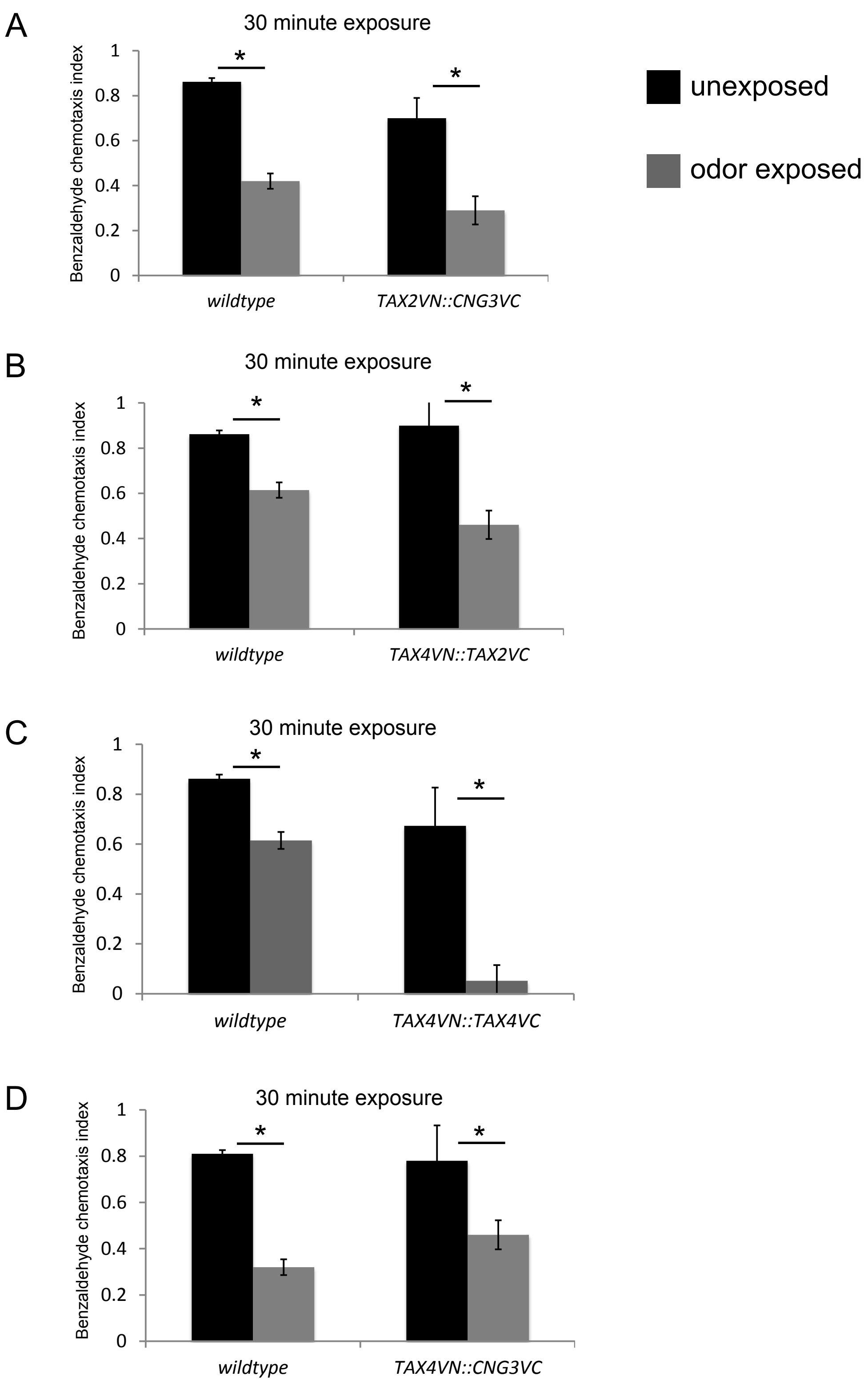
A
30 minute exposure
Benzaldehyde chemotaxis index
1
0.8
0.6
0.4
0.2
0
*
*
wildtype
TAX2VN::CNG3VC
unexposed
odor exposed
B
30 minute exposure
Benzaldehyde chemotaxis index
1
0.8
0.6
0.4
0.2
0
*
*
wildtype
TAX4VN::TAX2VC
C
30 minute exposure
Benzaldehyde chemotaxis index
1
0.8
0.6
0.4
0.2
0
*
*
wildtype
TAX4VN::TAX4VC
D
30 minute exposure
Benzaldehyde chemotaxis index
1
0.8
0.6
0.4
0.2
0
*
*
wildtype
TAX4VN::CNG3VC

Figure S4

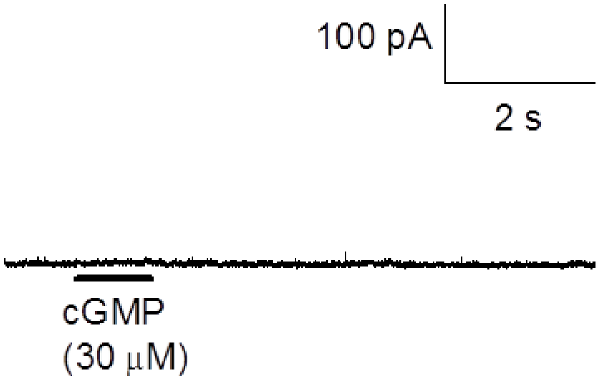

Supplement: Supplementary file 1 — Supplementary Figures [file 41598_2017_126_MOESM1_ESM.pdf]
